# Hyaluronic Acid Ultra-Porous Scaffolds Reinforced with Low Quantities of Graphene Oxide: Influence on the Delivery of Curcumin and Bacterial Inhibition

**DOI:** 10.3390/nano15100735

**Published:** 2025-05-14

**Authors:** Sandra Fuster-Gómez, Alberto J. Campillo-Fernández

**Affiliations:** Centre for Biomaterials and Tissue Engineering (CBIT), Universitat Politècnica de València, 46022 Valencia, Spain; sanfusgo@etsii.upv.es

**Keywords:** tissue engineering, hyaluronic acid, graphene oxide, curcumin, drug release

## Abstract

In this study, the influence of (i) the degree of crosslinking and (ii) incorporating small amounts of graphene oxide—up to 0.2% by mass—into the matrix of ultra-porous hyaluronic acid scaffolds was analyzed in relation to their physicochemical and mechanical properties. Also studied was the effect of incorporating graphene oxide on the release profile of curcumin, a hydrophobic molecule of interest in tissue engineering, plus the potential antibacterial activity of graphene oxide against *Escherichia coli*, *E.coli*. This paper describes the development of a hybrid ultra-porous material composed of crosslinked hyaluronic acid and graphene oxide, representing a significant advance in the field of nanomedicine. The resulting material enables dual control over the release kinetics of curcumin, a compound with high pharmacological potential and neuroprotective properties.

## 1. Introduction

Tissue engineering aims to repair and regenerate tissues using biomaterials that provide a three-dimensional environment essential for promoting cell adhesion, migration, and proliferation [1]. This process can be improved through advanced systems that combine biomaterials with bioactive molecules with properties that can enhance regeneration and cellular activity after release near the lesion to avoid the potential systemic side effects of traditional systems.

In this field, the functionality of hyaluronic acid (HA) offers a wide range of applications; hyaluronic acid is a glycosaminoglycan polysaccharide that plays a fundamental role as a structural component in the formation of the extracellular matrix (ECM) of many tissues. This is due to its interactions with proteoglycans or collagen, especially in connective tissues, but also in nervous tissue and epithelia, presenting properties of biocompatibility and biodegradability as well as excellent biological properties [2]. Hyaluronic acid is synthesized on the inner cell membrane by hyaluronan synthases, a class of transmembrane proteins [3].

Hyaluronic acid has been shown to be able to interact with different cell surface receptors, leading to the activation of intracellular signaling pathways and the induction of proliferative and migratory responses [4], as well as the regulation of immune and inflammatory responses and vascularization [4,5]. The presence of reactive groups in its structure also allows for functionalization or modification to form hydrogels [6] whose porous structure and excellent biocompatibility provide physical support for cell adhesion and proliferation [7].

Hydrogels can be defined as systems composed of one or more hydrophilic polymers whose chains are crosslinked through physical or chemical interactions to form a three-dimensional network. They are primarily characterized by their ability to absorb large amounts of aqueous fluids from the surrounding environment and swelling until reaching equilibrium. Due to the crosslinked nature of the polymer chains, hydrogels can absorb water without dissolving, while their high water content provides an ideal environment for cell survival. The degree of crosslinking plays a critical role in determining the porous structure and swelling behavior of the hydrogel.

Hydrogels are among the most frequently used systems in tissue engineering due to their ability to simulate the native extracellular matrix and have also served as a support matrix for drug administration [8]. The kinetics of drug release of HA hydrogels is mainly controlled by the diffusion mechanism, which can be modulated by the degree of crosslinking [9,10] or incorporating a filler material to form nanocomposite hydrogels to compensate for some of their limitations in biomolecule release [7].

Among these fillers for nanocomposite hydrogels, graphene and its derivatives have recently attracted the interest of researchers in the field of regenerative medicine, especially graphene oxide (GO), which has been widely studied as a potential therapeutic carrier due to its unique properties [11], including the ability to interact with hydrophobic molecules through π-π interactions due to its high surface area to volume ratio, sp2 carbon layer and the ability to interact with hydrophilic molecules through hydrogen bonding and electrostatic interactions by means of a variety of oxygen-containing functional groups [12]. This ability to interact with opposite types of molecules gives GO a high potential for functionalization and allows it to regulate the release pattern of a wide range of therapeutic molecules [13,14,15], enhancing its specific biological activity, biocompatibility and application to drug delivery [16]. GO has also attracted attention for its antibacterial properties, a desirable trait, as bacterial infection is now one of the leading causes of biomaterial implant failure [17].

There is a wide range of naturally occurring molecules in the context of tissue engineering that are being explored for their potential use of enhancing the performance of hydrogels including: curcumin (Cur), which has gained popularity due to its various pharmacological effects [18]. Curcumin (C_21_H_20_O_6_), the principal curcuminoid of the turmeric rhizome (*Curcuma longa*) contains a polyphenolic structure and presents a wide pharmacological potential due to its anti-inflammatory, antioxidant and its ability to interfere with different cellular pathways [19]. These pharmacological properties have been used against a plethora of cancers [20] and in regenerative medicine for wound healing [21], and musculoskeletal [22], bone [23] and cartilage [24] regeneration. Curcumin has also been proven to have a neuroprotective capacity, which has given rise to its investigation in lesions affecting the nervous system, as it provides a favorable environment for axonal recovery [25]. In previous research, it has been shown that curcumin can improve the oxidative damage associated with spinal cord injuries (SCI) due to its antioxidant capacity to react and eliminate lipid radicals from reactive oxygen species (ROS) in cell membranes [26]. It has also been shown that it can reduce apoptosis as well as improve neurite growth and cellular migration after SCI [27] and can promote the production of BDNF, playing a beneficial role in neurodegenerative diseases [28].

Despite the large number of studies on the benefits of using curcumin, its translation to the clinic and use as an effective pharmaceutical lead has proven somewhat challenging, due to its low bioavailability, poor solubility in water and short biological half-life [29,30,31], so that there is a real need for improved delivery platforms such as nanocomposite hydrogels.

To address these issues, the present study sought to develop a hyaluronic acid ultra-porous scaffold reinforced with low quantities of graphene oxide, which was then loaded with curcumin, a molecule of interest in the field of tissue engineering. A thorough physicochemical characterization of the HA-GO hydrogel developed was then conducted and the effect of GO concentration on bacterial growth and curcumin release profile was evaluated. Incorporating GO was shown to enhance the properties of the scaffold and increase the amount of drug that could be loaded. The combination of the advantageous traits of both components—hydrophilicity and biocompatibility of the HA hydrogel, along with the drug affinity of GO—renders the developed scaffold a promising resource for researchers and clinicians in the future.

## 2. Materials and Methods

### 2.1. Synthesis of HA and HA/GO Hydrogels

Hyaluronic acid materials were synthesized following the method proposed by Ortuño-Lizarán et al. [32], introducing modifications such as adding varying contents of graphene oxide (GO) and the amount of crosslinking agent in the reaction to study their effects on the materials and the drug release profile of the loaded compound.

Hyaluronic acid sodium salt from Streptococcus equi, HA, 1.5–1.8 MDa 53747, (Merck KGaA, Darmstadt, Germany) solutions at 5% wt/wt were prepared in a 0.2 M sodium hydroxide, S8045 (Merck KGaA, Germany) aqueous solution, into which varying amounts of graphene oxide (Graphenea S.A., Gipuzkoa, Spain) were dispersed. The graphene oxide underwent a pre-treatment of homogenization in the aqueous phase by sonication using graphene oxide powder as the starting material, as previously described [33]. Briefly, GO powder was dispersed in 1 mg/mL Milli-Q water (Merck KGaA, Germany) and sonicated for 2 h using a Bandelin Sonopuls HD3200 (Bandelin electronic GmbH & Co. KG, Berlin, Germany) equipped with a TT13FZ tip (50 W, 500 ms pulse cycle). The dispersion was centrifuged at 14,000× *g* rpm for 25 min at Eppendorf 5804R (Eppendorf SE, Hamburg, Germany), after which the supernatant was removed, and the pellet was vacuum-dried at 40 °C for 24 h. The amounts of graphene oxide in the HA/NaOH stock solution were calculated in each case to achieve a final GO fraction of 0, 0.1, and 0.2% wt/wt, relative to the amount of HA. The solutions were stirred for 24 h and then subjected to ultrasonication for 1 h to achieve a homogeneous dispersion of the GO.

Divinyl sulfone, DVS, V3700 (Merck KGaA, Germany) was used as a crosslinking agent at a molar ratio of 8:10 and 9:10 relative to the hyaluronic acid (0.8 and 0.9 moles of DVS per mol of HA repetition unit). The HA-DVS and HA-GO-DVS solutions were stirred for 10 s to achieve homogenization and were placed in 7 cm diameter Petri dishes by means of a micropipette to achieve a uniform sample thickness. The samples were left to gel at room temperature and subsequently frozen at −20 °C for a minimum of 2 h and at −80 °C for at least 30 min. The samples were lyophilized (LyoQuest-85, Syntegon Telstar, S.L.U., Barcelona, Spain) at <10 Pa overnight (O/N) to obtain a porous structure through the sublimation of the crystals formed during freezing.

**Figure 1:**
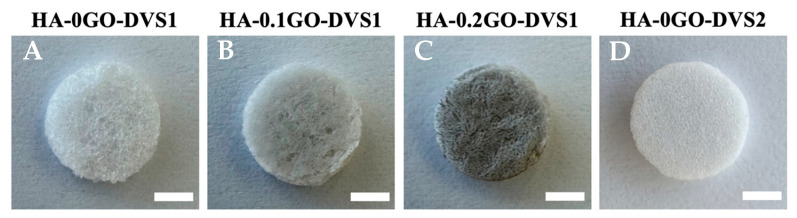
Representative macroscopic photographs of HA scaffolds reinforced with different GO concentrations and different degrees of crosslinking. (**A**) HA-0GO-DVS1 (no GO and DVS molar ratio of 8:10); (**B**) HA-0.1GO-DVS1 (0.1% wt/wt GO and DVS molar ratio of 8:10); (**C**) HA-0.2GO-DVS1 (0.2% wt/wt GO and DVS molar ratio of 8:10) and (**D**) HA-0GO-DVS2 (no GO and DVS molar ratio of 9:10). Photographs were taken after washing and freeze-drying. Scale bar 2 mm.

The films obtained were demolded and hydrated in distilled water (H_2_Od) before being punched into discs 6 mm in diameter and washed for 2 h in H_2_Od to remove any potential residual NaOH and DVS, followed by overnight lyophilization (Figure 1).

### 2.2. Characterization of HA and HA/GO Hydrogels

#### 2.2.1. Morphological Characterization by Scanning Electron Microscopy (SEM)

The morphology of the interior of the samples was studied by scanning electron microscopy (SEM) on a JSM-3600 microscope (JEOL, Ltd., Tokyo, Japan) after overnight lyophilization, to prevent potential interference caused by the evaporation of residual water and were cut transversely using liquid nitrogen to observe the pore distribution and morphology. To ensure sample conductivity, a platinum coating was applied at a voltage of 15 kV, and the observation voltage used was 3 kV.

#### 2.2.2. Swelling Ratio

The degree of swelling or water absorption capacity of the synthesized HA and HA/GO discs was determined at equilibrium by performing a swelling test by comparing the dry and swollen weights. The disks, lyophilized overnight, were weighed in the dry state and then hydrated for 2 h at room temperature in distilled water until equilibrium to obtain their swollen weight measured in three replicates (n = 3) of each sample type.

The swelling ratio was calculated by Equation (1), where w refers to the weight of the discs measured in both the dry (w_D_) and swollen (w_S_) states.

(1) $$Swelling\;ratio=\frac{w_{S}-w_{D}}{w_{D}}\cdot 100$$

#### 2.2.3. Density and Porosity

The sample density and porosity were determined using a gravimetric method based on Archimedes’ principle. Three replicates of each sample were weighed on a Mettler Toledo AE 240 (Mettler-Toledo GmbH, Zurich, Switzerland) precision balance with a readability of 0.01 mg, equipped with a Mettler ME 33360 (Mettler-Toledo GmbH, Switzerland) kit accessory to determine the density.

The dry weight of the discs was first recorded (w_D_), after which the discs were placed in BD Vacutainer tubes connected to a vacuum pump RZ 2 (Vacuubrand GMBH + CO KG, Wertheim, Germany) and filled with n-octane to replace the air inside the pores with the solvent. After removing excess n-octane from the surface of the samples with absorbent paper, the weight of the discs with n-octane in their pores was measured (w_P_). Finally, the samples with n-octane in the pores were fully immersed in the solvent to obtain the weight of the scaffolds submerged in n-octane (w_L_). Density and porosity measurements were repeated on 3 replicates of each scaffold type.

The density was determined by applying Archimedes’ principle:

(2) $$\rho_{s}=\frac{w_{D}}{w_{D}-w_{L}}\cdot \rho_{0}$$

where ρ_S_ is the sample density and ρ_0_ is the density of n-octane.

Porosity, π, as a percentage, is calculated using Equation (3):

(3) $$\pi \left(\%\right)=\frac{V_{pores}}{V_{total}}\times 100=\frac{w_{P}-w_{D}}{w_{P}-w_{L}}\times 100$$

#### 2.2.4. Thermomechanical Analysis (TMA) or Mechanical Testing

Compression tests were performed in both the dry and swollen states in the longitudinal direction of the synthesized scaffolds to evaluate the effect of graphene oxide addition and the increased proportion of the cross-linking agent on the mechanical properties. The scaffolds were punched into smaller 3 mm-diameter discs to achieve suitable dimensions for the equipment. All the measurements were conducted at a speed of 100 mN/min up to a load of 1500 mN. Dry state measurements were carried out at room temperature (25 °C), while the swollen state measurements were done in water at 37 °C using the Seiko EXSTAR TMA/ss6000 dilatometer (Hitachi, Tokyo, Japan). Ten replicates were analyzed for each type of sample and the mean and standard deviation were calculated. The compression modulus, or Young’s modulus, was determined as the slope of the stress–strain curve in the elastic region.

#### 2.2.5. Fourier-Transform Infrared Spectroscopy (FTIR)

Fourier-transform infrared spectroscopy (FTIR) was performed on a Bruker Alpha FTIR spectrometer (Billerica, MA, USA) in absorbance mode to identify the functional groups present in the samples. The spectra were obtained by averaging 24 scans in the mid-infrared region at a resolution of 4 cm^−1^, ranging from 400 to 4000 cm^−1^.

### 2.3. Drug Loading on HA and HA/GO Scaffolds

Curcumin, Cur, C1386 (Merck KGaA, Germany) was selected as the model drug. A 1 mg/mL solution in ethanol (EtOH) was prepared to load the drug into the HA samples.

The loading volume of the HA and HA/GO samples was 10 µL. The calibration curve was prepared from standard drug solutions, with concentrations ranging from 0.10 to 0.0005 mg/mL.

#### In Vitro Drug Release

The release profile of the HA and HA/GO scaffolds loaded with the drug was studied by placing them in vials with 1 mL of phosphate-buffered saline (PBS) at 37 °C and incubating them with agitation. 1 mL aliquots (total extraction) were taken at predetermined time intervals to determine the concentration, replacing the extracted medium with a fresh medium to maintain constant volume. The absorbance of the extracted release medium was analyzed at 426 nm on a ND-100 UV-Vis Spectrophotometer (Thermo Scientific, Waltham, MA, USA). The release assay was performed in triplicate for each condition studied (n = 3).

The results of the in vitro release assay were analyzed and compared with the kinetic model proposed by Fick’s law (Equation (4)), which describes the diffusion of the drug contained in the hydrogel matrix into the release medium:

(4) $$\frac{\partial c_{1}}{\partial t}=D\frac{\partial^{2}c_{1}}{\partial x^{2}}$$

where $c_{1}$ is the concentration of the substance diffused into the medium, t is the time, x is the spatial direction normal to the section, and D is the diffusion coefficient (proportionality constant). Equation (4) can be approximated in the case of the diffusion through a sheet with thickness l, for short times corresponding to $\frac{m_{t}}{m_{\infty }}<0.5$ by the equation $\frac{m_{t}}{m_{\infty }}=\frac{4}{\pi }\frac{\sqrt{t\cdot D}}{l^{2}}$, where $m_{t}$ and $m_{\infty }$ are the drug release at time t and at equilibrium, respectively [34]. The drug release change $\frac{m_{t}}{m_{\infty }}$ must therefore be a linear function of the square root of time in the initial stage of the desorption process. The slope of the $\frac{m_{t}}{m_{\infty }}$ vs. $\sqrt{t}$ calculates the values of D.

The release data in aqueous medium were fitted by Ritger–Peppas’ model to compare its fit goodness (Equation (5)):

(5) $$\frac{M_{t}}{M_{\infty }}=Kt^{n}$$

where K is related to the velocity of the drug release, n to the mechanism of drug release (diffusion, diffusion and matrix relaxation, matrix erosion/dissolution) and $M_{t}$ and $M_{\infty }$ are the drug mass released into the medium at time t and the total mass of the released drug, respectively.

### 2.4. In Vitro Antibacterial Activity Assays

#### 2.4.1. Sample Sanitization

Before testing any antibacterial activity, hyaluronic acid (HA) and hyaluronic acid-graphene oxide (HA-GO) scaffolds were sanitized by immersion for 2 h in 70° ethanol, ET0002025P (Scharlab, Barcelona, Spain) followed by a 10 min immersion with 50° and 30° ethanol. The samples were then immersed in sterile Milli-Q water for 10 min to remove any ethanol residue (in a laminar flow cabinet to ensure a sterile environment). Finally, the samples were lyophilized under sterile conditions to remove any excess water.

#### 2.4.2. Bacterial Strain Preparation

Gram-negative *Escherichia coli* (*E. coli*, CECT 434) was subcultured (Passage 1) on LB-agar 1083 (Condalab, Madrid, Spain) and incubated at 37 °C for 24 h. For the inoculum preparation, 4 to 5 single colonies were selected from the LB-agar plate and resuspended in 1 mL of sterile saline water 0.9% *w*/*v* NaCl, SO0225005P (Scharlab, Spain). The inoculum’s absorbance was measured on a spectrophotometer Nanodrop One (Mettler-Toledo S.A.E., Zurich, Spain) at an optical density of 600 nm (OD) to ensure the correct seeding cell density.

#### 2.4.3. Disk-Diffusion Susceptibility Test

The antibacterial activity of the HA and HA-GO scaffolds was evaluated by the Kirby-Bauer disk diffusion susceptibility test method [35]. Briefly, the *E. coli* inoculum was evenly spread onto Mueller-Hinton agar pH 7.25, MH-agar, 70192 (Merck KGaA, Germany) with the help of a sterile cotton swab and sterile tweezers to properly distribute them on the inoculated MH-agar. A 10 μg Ampicillin disk, CT0003B (Fisher Scientific, Loughborough, UK) was placed on each MH-Agar plate as a positive control. The MH-agar plates were then incubated at 37 °C for 24 h, after which the plates were photographed and the inhibition zones measured by a Vernier calliper.

#### 2.4.4. Colonization Assay

As no inhibition halo was seen on the MH-agar plates, after 24 h at 37 °C in contact with *E. coli* bacterial lawns on the MH-Agar plates to determine whether bacteria had colonized the scaffolds, they were placed in 15 mL of LB broth and incubated under aerobic conditions at 37 °C in a shaker incubator at 250 rpm for 24 h. The OD at 600 nm was then measured by spectrophotometer Nanodrop One (Mettler-Toledo S.A.E., Barcelona, Spain). The average and standard deviation of the OD600 nm readings (n = 4) was calculated and plotted against the GO concentration.

### 2.5. Statistical Analysis

Data were expressed as mean ± standard deviation (SD). Statistical analysis was performed on GraphPad Prism 9.4.0 (Boston, MA, USA). Data normality was assessed by the Shapiro–Wilk test. One-way (porosity, density, swelling ratio and antimicrobial activity), or the two-way ANOVA test (thermomechanical analysis) was used to conduct the statistical analysis. Multiple comparisons were performed using Tukey’s post hoc analysis if significant differences were determined and statistical significance was accepted at the probability level * *p* < 0.05; ** *p* < 0.01; *** *p* < 0.001; **** *p* < 0.0001.

## 3. Results and Discussion

### 3.1. Morphology of HA Films

**Figure 2:**
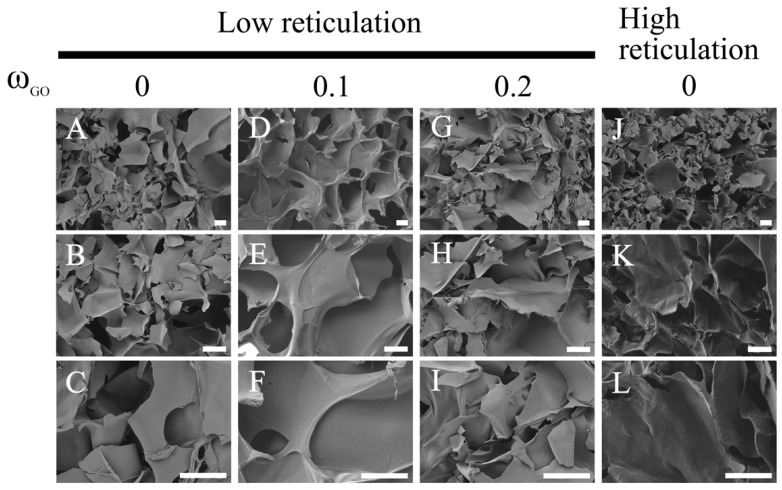
Morphology of HA with different DVS and GO contents. Representative High-resolution scanning electron microscopy (SEM) images show HA films with different degrees of crosslinking (**A**,**J**) and different amounts of GO (**D**,**G**). (**B**,**C**) are a magnification image of (**A**); (**E**,**F**) are a magnification image of (**D**); (**H**,**I**) are a magnification image of (**G**), (**K**,**L**) are a magnification image of (**J**). Scale bar 30 μm.

Figure 2 shows the internal structure of the scaffolds at different magnifications, based on the DVS and GO content. As can be seen, the formation of cavities due to sublimation of the ice crystals is not affected by either parameter and provides highly ultra-porous and well-interconnected structures.

The extent of crosslinking has no impact on the pore size, which is instead determined by crystal growth, which can clearly b seen in the case of scaffolds fabricated using a template and subsequent dissolution: pore size would be dictated by the template size rather than the degree of crosslinking but in this case is determined by the crystal growth, which is independent of the DVS and GO quantities.

Pore sizes ranging between 50 and 100 microns can be seen: the broad pore size distribution in the lyophilized hyaluronic acid-based hydrogels can be attributed to the nature of the freeze-drying process, in which the pores are generated by the sublimation of ice crystals formed during the freezing step prior to lyophilization. The size and distribution of these ice crystals—and, consequently, the resulting pores—are strongly influenced by the freezing temperature, cooling rate and even the mold design, leading to heterogeneous pore sizes [36], although the observed pore size makes these substrates suitable for blood vessel ingrowth [37].

It should also be noted that no GO macroaggregates are visible, indicating good dispersion of GO within the HA matrix.

### 3.2. Porosity and Density

**Figure 3:**
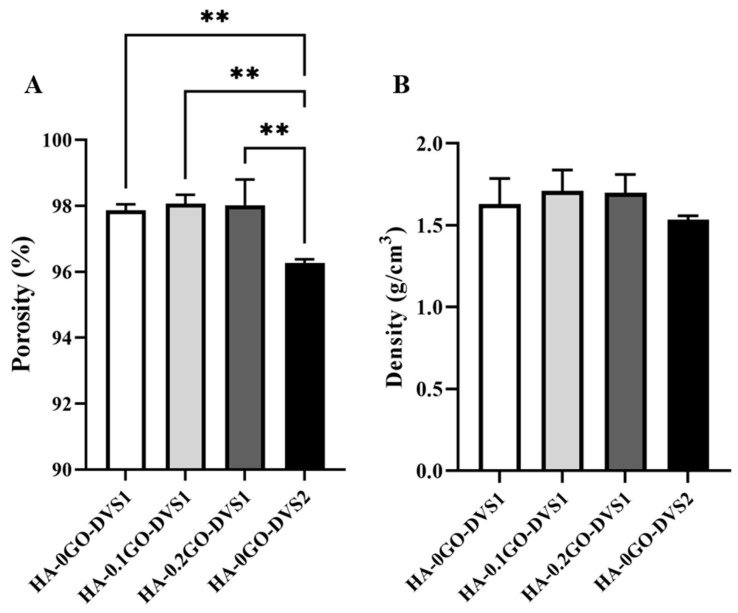
Porosity and density of HA discs with different DVS and GO contents. (**A**) porosity and (**B**) density were calculated by Archimedes’ principle using n-octane. Bars represent mean ± standard deviation calculated for each group (n = 3). Statistical differences were assessed by one-way ANOVA with Tukey’s test and are indicated by **, donating a *p*-value below 0.01.

Figure 3 shows the porosity (Figure 3A) and density (Figure 3B) of the scaffolds as a function of crosslinking agent and GO content. We found that the degree of crosslinking influences the scaffold’s porosity, increasing the crosslinker content reduced the overall sample porosity from 98% to 96% porosity (HA-0GO-DVS1 and HA-G0-DVS2, respectively). On the other hand, even though the density is reduced when crosslinking is increased from 1.63 g/cm^3^ to 1.53 g/cm^3^ density, no statistical differences were found, nor were there statistically significant difference in scaffold porosity or density in relation to their GO content.

### 3.3. Modulation of HA Swelling by Crosslinking

**Figure 4:**
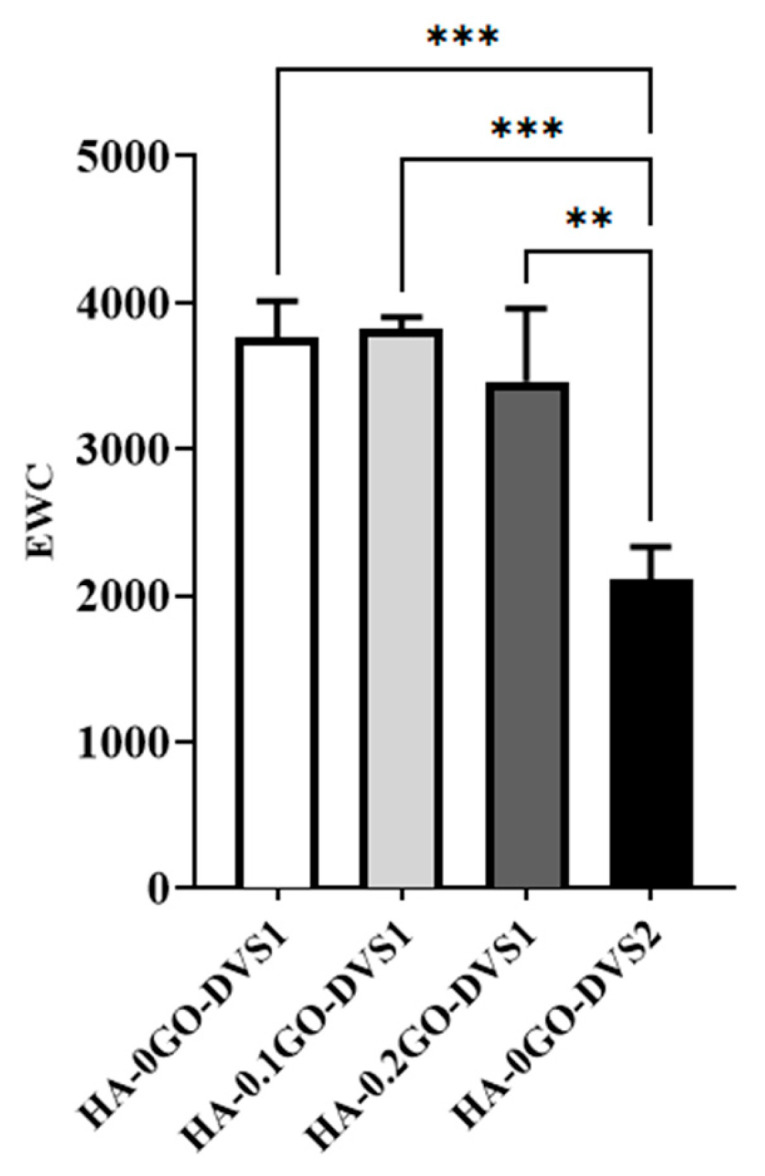
Equilibrium water contents (EWC) of HA films with different DVS and GO content. EWC was calculated after equilibration in liquid water as a function of the GO content, while crosslinking degree: referred to the dry mass of the samples (n = 3). Statistical differences were assessed by one-way ANOVA with Tukey’s test and are indicated by, ** or ***, denoting a *p*-value below 0.01 or 0.001, respectively.

Figure 4 shows that the amount of water absorbed is also influenced by the crosslinker content; with higher DVS the network becomes more restricted, resisting water entry and going from an initial value of 3761 to 2108 as the degree of crosslinking is increased. This is a classic effect that provides control of the swelling degree through the degree of crosslinking. While adding GO also seems to influence this degree to a lesser extent than crosslinking; swelling is seen to decrease with increasing GO content, although no statistical differences were found.

### 3.4. Fourier Transform Infrared Spectroscopy (FTIR)

**Figure 5:**
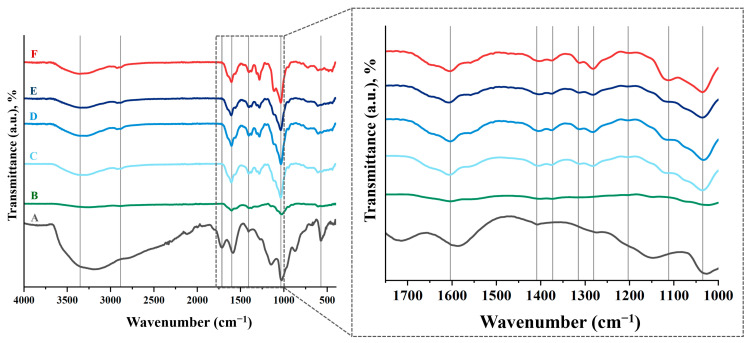
FTIR spectra of HA films with different DVS and GO content. (A) GO, (B) HA powder, (C) HA-0GO-DVS1, (D) HA-0.1GO-DVS1, (E) HA-0.2GO-DVS1 and (F) HA-0GO-DVS2. Lines indicate the characteristic peaks of functional groups found.

Figure 5 shows the FTIR spectra of the neat HA and the fabricated HA-GO samples, presenting a characteristic band that appeared at 3000–3600 cm^−1^ in the high frequency area, attributed to the stretching mode of the O-H bond, which reveals the presence of hydroxyl groups in both HA and graphene oxide.

As DVS, which is used as a crosslinker, can have a harmful effect on living organisms due to its toxicity [38], it is advisable to check that no unreacted DVS residues remain after the scaffold manufacturing process. The most characteristic peak occurs at a wavelength of 1300–1350 cm^−1^, due to stretching of the S=O double bond. It can be seen that this peak does not exist in the scaffolds, just as it does not occur in the starting HA powder (Figure 5). This suggests that the manufacturing process is effective and unreacted DVS residues are eliminated by adequate washing of the samples.

### 3.5. Thermomechanical Analysis

**Figure 6:**
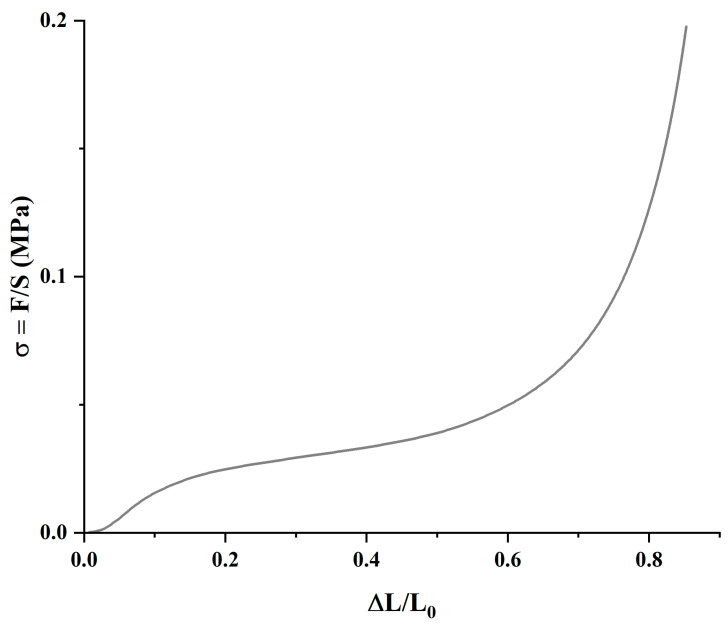
Representative mechanical compression regions of the HA-0GO-DVS1 sample in air.

The materials’ mechanical compression behavior follows a typical pattern, as shown in Figure 6. Three clearly differentiated regions can be identified: the first is due to the mechanical properties of the porous material from the start until the pores begin to collapse; the second region, characterized by a broad plateau, represents the progressive collapse of the pores; while the third reflects the material’s response after the pores have fully collapsed.

**Figure 7:**
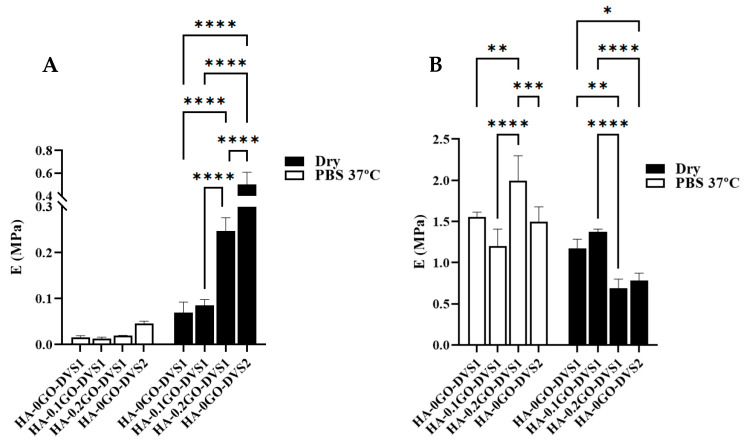
Elastic moduli of HA films with different DVS and GO contents. Elastic moduli corresponding to (**A**) the porous part and (**B**) the collapsed part determined by compression test under air conditions and immersed in PBS at 37 °C (n = 10). Statistical differences were assessed using two-way ANOVA with Tukey’s multiple comparisons test and are indicated by *, **, *** or ****, denoting a *p*-value below 0.05, 0.01, 0.001 or 0.0001, respectively.

It is important to evaluate the mechanical behavior of materials under both dry and hydrated conditions, simulating storage and implantation environments, respectively. Figure 7 displays the mechanical moduli obtained in the first phase, E_porous_, (A) and in the mechanical moduli once the scaffolds collapsed, E_collapsed_, (B), both in air and immersed in PBS fluid. When analyzing the porous modulus, it is evident that both the degree of cross-linking and the GO content influence the values. Clearly, both in air and in PBS, the modulus increases as DVS increases (classic behavior), and it also increases with the GO content, indicating an interfacial interaction between both components.

In the case of samples immersed in PBS, Figure 7A, increasing the degree of crosslinking increases the modulus, although the differences are not statistically significant. These low values are attributed to the scaffolds’ extremely ultra-porous structure. However, the effect of adding both GO and crosslinker is clearly seen in the porous modulus: the sample with a higher DVS content shows significant differences with the rest of the samples, and except for the sample without GO and with the lowest content, all show statistically significant differences. Incorporating GO thus has a reinforcing effect on the compression modulus. However, the results become chaotic when analyzing the collapsed modulus: differences occur between samples but without any correlation with the amount of GO or DVS.

Using the classical theory of rubber elasticity [39]

$$∆G_{elastic}=\frac{1}{2}Rn_{c}T\left(\lambda_{x}^{2}+\lambda_{y}^{2}+\lambda_{z}^{2}-3\right)$$

$n_{c}$ being the number of chain moles and $\lambda_{i}=\frac{L_{i}}{L_{0}}$ the stretching ratios along the three orthogonal principal directions. As $\lambda_{x}\lambda_{y}\lambda_{z}=1$ (elastomer incompressibility) and considering uniaxial deformation it yields to:

$$∆G_{elastic}=\frac{1}{2}Rn_{c}T\left(\lambda^{2}+\frac{2}{\lambda }-3\right)$$

Then, force $f=\frac{\partial ∆G}{\partial L}=\frac{Rn_{c}T}{L_{0}}{(\lambda -\lambda }^{-2})$, and tension σ=fA=fV2L=RncTV2λ2−λ−1=ρRTMcλ2−λ−1, $V_{2}$ being the volume occupied by the polymer.

Finally, elastic modulus, $E=\underset{\lambda \to 1}{\lim}\frac{d\sigma }{dL}\frac{\partial ∆G}{\partial \lambda }=3\frac{n_{c}}{V_{2}}RT=3\frac{\rho }{M_{c}}RT$, which relates the elastic modulus with the $\frac{n_{c}}{V_{2}}$ parameter and gives the structural characteristic parameter (number of polymer chain moles per polymer volume).

Considering that the materials are scaffolds, the E modulus obtained in mechanical experiment, $E_{porous}$, should be converted to $E_{non-porous}$, through $E_{porous}=E_{non-porous}(1-\frac{V_{porous}}{V_{2}+V_{porous}})$.

As clearly shown in Table 1, both GO and DVS parameters constrain the HA network. The number of polymer chain moles per polymer volume decreases drastically when comparing the degree of crosslinking (3730 vs. 1069 mol/cm^3^), which is consistent with the water absorption capacity (see Figure 4). Additionally, GO hinders chain mobility, increasing the elastic modulus, thereby acting as a positive mechanical reinforcement element for the scaffolds.

### 3.6. Curcumin Release

Although curcumin is widely recognized for its promising pharmacological properties, which have made it an attractive candidate in the field of tissue engineering [18], its clinical application is still limited due to inherent drawbacks, such as poor aqueous solubility, rapid metabolic degradation and low systemic bioavailability [31]. Given these limitations, it becomes essential to develop delivery systems capable of enhancing its stability and sustained release. In this work, we investigated the release behavior of curcumin from hyaluronic acid-based scaffolds, specifically evaluating how variations in crosslinking density and GO content influence the release kinetics. This approach aims to determine whether the designed scaffolds can serve as an effective platform for delivering curcumin, potentially overcoming its known limitations.

**Figure 8:**
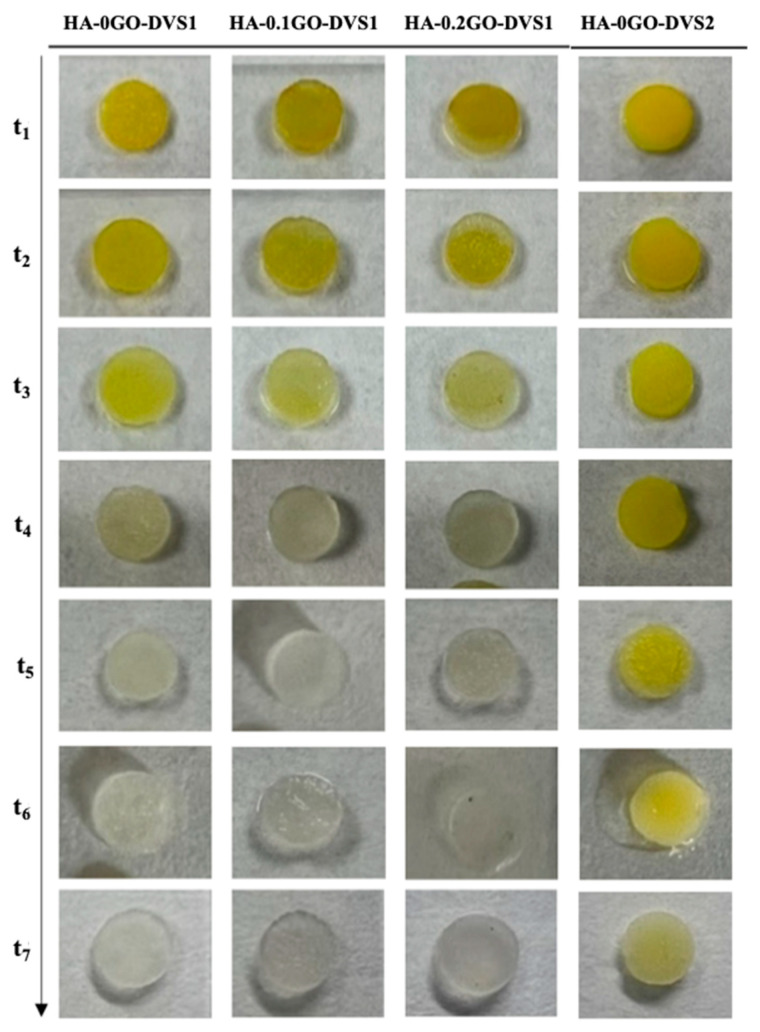
Representative macroscopic images of HA films with different DVS and GO contents. Images illustrate the curcumin release of the different scaffolds at the studied time points after 21 days in PBS. As time progresses, the characteristic yellow color of curcumin disappears.

Figure 8 shows the release process of curcumin from different polymeric matrices. Curcumin’s characteristic yellow is quite pronounced at short times and decreases as time increases due to the release of the molecule into the medium. It can also be seen that at long times, part of the loaded curcumin is retained (clearly seen in the sample with the highest degree of cross-linking), indicating a strong interaction with the polymeric matrix.

**Figure 9:**
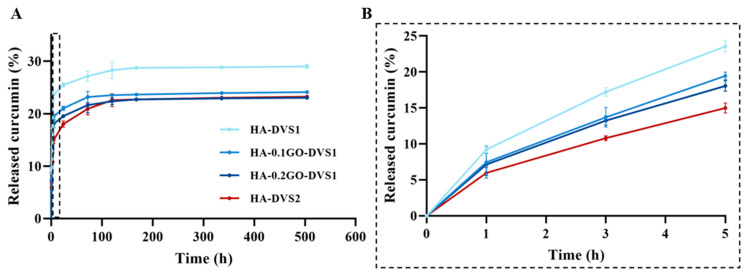
In vitro drug release profiles of curcumin from HA films with different DVS and GO contents in PBS at 37 °C (**A**). Curcumin release profiles at time region between 0 and 5 h to better visualize drug release curves of samples (**B**). Results are represented as mean ± standard deviation calculated for each time studied (n = 3).

The release kinetics of curcumin from the polymeric matrix of the different samples can be seen in Figure 9 and its adjustment to Fick’s law and Ritger-Peppas model in Supplementary Figures S1 and S2, respectively. It is apparent that the curcumin released in the medium ranges between 22% and 30%, depending on crosslinker content and GO. The HA network without GO and with less crosslinking has the characteristic of having the highest degree of released molecules and releasing them faster, as clearly seen in Table 2, from the calculation of the desorption coefficients.

The first effect is seen when increasing the degree of crosslinking: the network constricts, and both the amount and rate of curcumin desorption are reduced. This can be explained by the fact that curcumin retains primarily by physical entrapment. The hydrogel absorbs the curcumin-containing solution, and the drug becomes confined within the porous network. These interactions are mostly weak and physical in nature, with no specific affinity between HA and curcumin with diffusion as the dominant driving mechanism. Greater crosslinking results in slower curcumin release, likely due to reduced matrix porosity and diffusivity.

On the other hand, when GO is added, the desorbed molecule and its rate decrease monotonically, i.e., the more the GO the less the desorbed amount of molecule and rate. This phenomenon could be attributed to the network is constrained by including GO lamellae and also due to the fact that when graphene oxide (GO) is incorporated into the HA matrix, additional interaction mechanisms come into play. GO offers a high surface area and sp^2^-hybridized carbon domains capable of forming π–π stacking interactions with the aromatic rings of curcumin [12], which increase the affinity of curcumin for the scaffold.

The effect of adding GO is equivalent in absolute terms to increasing the amount of crosslinker, since the accumulated release is very similar in both cases. However, it should be highlighted that although the amount of curcumin released is similar in the samples without GO and with high DVS content (HA-0GO-DVS2) when compared to samples with higher amounts of GO (HA-0.2GO-DVS1), the release rate decreases as GO content increases.

As can be seen in Table 2, once again, the diffusion coefficients confirm that cross-linking the network induces a significant change in the curcumin diffusion rate, that reduces the diffusion coefficient by 50%, while the effect of adding GO also contributes reducing it, indicating the greater difficulty for its release.

The Ritger–Peppas model, $\frac{M_{t}}{M_{\infty }}=Kt^{n}$, was used to analyze the release kinetics of each sample, yielding parameters K and n. which describe the release rate and mechanism from time interval 0 < t < 5 h, corresponding to approximately to a 60% release (Table 3).

As previously mentioned, the results confirm that the diffusion rate is significantly reduced, particularly when the polymeric network is restricted with a higher degree of cross-linking and, to a lesser extent, through the addition of GO. The exponential parameter, n, which represents the release mechanism and has a value around 0.5 (Fickian model), indicates a release mediated by molecular diffusion, while values between 0.5 and 1 suggest a combination of diffusion with structural relaxation of the polymeric matrix, as in the case in the studied system, in which all the n parameters range from 0.55 to 0.58.

### 3.7. Antimicrobial Activity of Graphene Oxide Hyaluronic Acid Scaffolds

GO bactericidal activity is thought to be mediated by the physical rupture of the bacterial cell wall on close contact with GO shards and its oxidative stress, which leads to lipid peroxidation [17,40,41,42]. It was therefore thought necessary to study the antimicrobial activity of the GO concentrations used in the HA-GO scaffolds, for which bacterial strain *E. coli* was selected, as it is commonly associated with healthcare-related bacterial infections [43,44].

**Figure 10:**
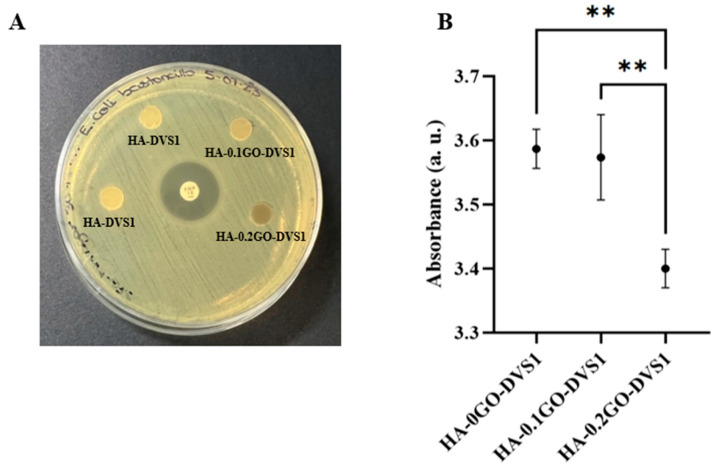
Antimicrobial activity of hyaluronic acid and graphene oxide hyaluronic acid scaffolds. (**A**) *E. Coli* antimicrobial susceptibility testing of HA and HA-GO discs and 10 μg antibiotic disc (Ampicillin) as control using Kirby–Bauer disk diffusion susceptibility test. (**B**) Absorbance at 600 nm after incubation of each scaffold on LB liquid for 24 h at 37 °C culture (n = 4). Statistical differences were assessed using one-way ANOVA with Tukey’s test and are indicated by **, indicating a *p*-value below 0.01.

The antibacterial activity of HA-GO scaffolds was assessed by the Kirby-Bauer disk diffusion susceptibility test. The results, as shown in Figure 10A, indicate, at least to the naked eye, that no inhibitory zone was detected, regardless of the GO concentration used in the HA-GO scaffolds. To determine whether our HA-GO scaffolds had any effect on the bacterial strain used, we incubated the scaffolds in LB liquid agar for 24 h at 37 °C and measured the absorbance at 600 nm. This allowed us to evaluate the bacteria that had been in close contact with the scaffolds and assess the actual effect of the GO. Figure 10B shows the absorbance measured against GO concentration. The absorbance, directly related to bacterial cell concentration decreased as the GO concentration increased, implying a decrease in bacterial growth. Statistical differences were found between all the samples, thus indicating the antibacterial effect of small quantities of GO.

While OD600 measurements do not distinguish between viable and non-viable cells, they provide a widely accepted indirect indicator of bacterial biomass and metabolic activity. These results indicate that the presence of GO in the scaffolds may hinder bacterial colonization or growth, even in the absence of classical inhibition halos. Nevertheless, we must acknowledge that this method has inherent limitations, and further studies involving live/dead bacterial staining or colony-forming unit (CFU) quantification would provide more direct evidence of antibacterial efficacy. This inhibition of bacterial growth is a crucial aspect to consider for their future use in the field of tissue engineering.

## 4. Conclusions

This paper describes a study on the effects of crosslinking degree and the addition of small quantities of graphene oxide (GO) on ultra-porous hyaluronic acid scaffolds. The results show that the formation of cavities due to sublimation of ice crystals is not affected by either parameter, resulting in highly ultra-porous and well-interconnected structures. The degree of crosslinking influences polymer porosity, with higher crosslinking leading to decreased porosity. The addition of GO also affects the degree of swelling, but to a lesser extent than crosslinking.

The mechanical properties of the scaffolds are influenced by both crosslinking and GO content, while incorporating GO has a reinforcing effect on the compression modulus, which becomes evident when the material is porous.

The results of curcumin release showed that the amount of curcumin released ranged from 22% to 30%, depending on the crosslinking content and GO. The HA matrix without GO and with less crosslinking had the highest amount of released curcumin and was faster. The addition of GO reduced the release rate and amount of curcumin, suggesting an interaction between GO and curcumin. The diffusion coefficients confirmed that crosslinking and GO content affected the release kinetics. The Ritger–Peppas model revealed a Fickian diffusion mechanism, with a combination of diffusion and structural relaxation of the polymer matrix.

The results also show that the release of curcumin from HA matrices is influenced by both the degree of crosslinking and GO content. Adding GO reduces the release rate and amount of curcumin, suggesting potential applications in controlled drug delivery systems. These findings have important implications for the design of systems for the controlled release of bioactive compounds.

The study also analyzed the effect of HA-GO scaffolds on bacterial growth, using *E. coli* as the model organisms. The results show that the absorbance, directly related to bacterial cell concentration, decreases as the GO concentration increases, implying a decrease in bacterial activity. Overall, this study demonstrates the potential of ultra-porous hyaluronic acid scaffolds with tailored mechanical and physicochemical properties for tissue engineering applications, representing a significant advance in the field of nanomedicine. The resulting material enables dual control over the release kinetics of curcumin, a compound with a high pharmacological potential and neuroprotective properties. Further research will be needed to fully explore the potential of these scaffolds and optimize their properties for specific applications.

## Figures and Tables

**Table 1:** Structural characteristic parameters of the HA-GO scaffolds.

	E_porous_ (MPa)	Porosity (%)	E_non-porous_ (MPa)	$\frac{n_{c}}{V_{2}}$ (mol/cm^3^)
HA-0GO-DVS1	0.0690	97.87	3.25	3730
HA-0.1GO-DVS1	0.0850	98.07	4.43	3031
HA-0.2GO-DVS1	0.247	96.28	6.64	1982
HA-0GO-DVS2	0.477	96.52	14.31	1069

**Table 2:** Diffusion coefficients.

Sample	Diffusion Coefficient	
HA-0GO-DVS1	0.0311	base
HA-0.1GO-DVS1	0.0160	−49%
HA-0.2GO-DVS1	0.0247	−20%
HA-0GO-DVS2	0.0238	−23%

**Table 3:** Parameters of the Ritger–Peppas release model.

Sample	K	n	Release at t = 5 h (%)
HA-0GO-DVS1	0.0311	base	59
HA-0.1GO-DVS1	0.0160	−49%	64.1
HA-0.2GO-DVS1	0.0247	−20%	56.2
HA-0GO-DVS2	0.0238	−23%	56.4

## Data Availability

Data will be made available on request.

## Supplementary Materials

The following supporting information can be downloaded at: https://www.mdpi.com/article/10.3390/nano15100735/s1, Figure S1: Time dependence of curcumin release calculated for HA films with different DVS and GO content with the kinetic model proposed by Fick’s law: (a) HA-0GO-DVS1, (b) HA-0.1GO-DVS1, (c) HA-0.2GO-DVS1 and (d) HA-0G0-DVS2; Figure S2: Curcumin release data fitted to Ritger–Peppas’ model.

## Abbreviations

The following abbreviations are used in this manuscript:

HA	Hyaluronic acid
GO	Graphene oxide
DVS	Divinyl sulfone
Cur	Curcumin
TMA	Thermomechanical analysis
FTIR	Fourier-Transform Infrared Spectroscopy
LB	Lysogeny Broth
MH	Müller-Hinton
PBS	Phosphate-Buffered Saline
OD	Optical Density
