# Supplementary material for: Hyaluronic Acid Ultra-Porous Scaffolds Reinforced with Low Quantities of Graphene Oxide: Influence on the Delivery of Curcumin and Bacterial Inhibition
Source: Nanomaterials (Basel). 2025 May 14;15(10):735. doi: 10.3390/nano15100735 (PMC12114591; doi:10.3390/nano15100735)
Supplement: Supplementary file 1 [file nanomaterials-15-00735-s001.zip › nanomaterials-3558546-supplementary.pdf]

Supplementary

Hyaluronic Acid Ultra-Porous Scaffolds Reinforced with Low Quantities of Graphene Oxide: Influence on the Delivery of Curcumin and Bacterial Inhibition

Sandra Fuster-Gómez [1] and Alberto J. Campillo-Fernández [1,*]

1 Centre for Biomaterials and Tissue Engineering (CBIT), Universitat Politècnica de València, Valencia, 46022, Spain; sanfusgo@etsii.upv.es (S.F.G.)

* Correspondence: alcamfer@ter.upv.es (A.J.C.F.)

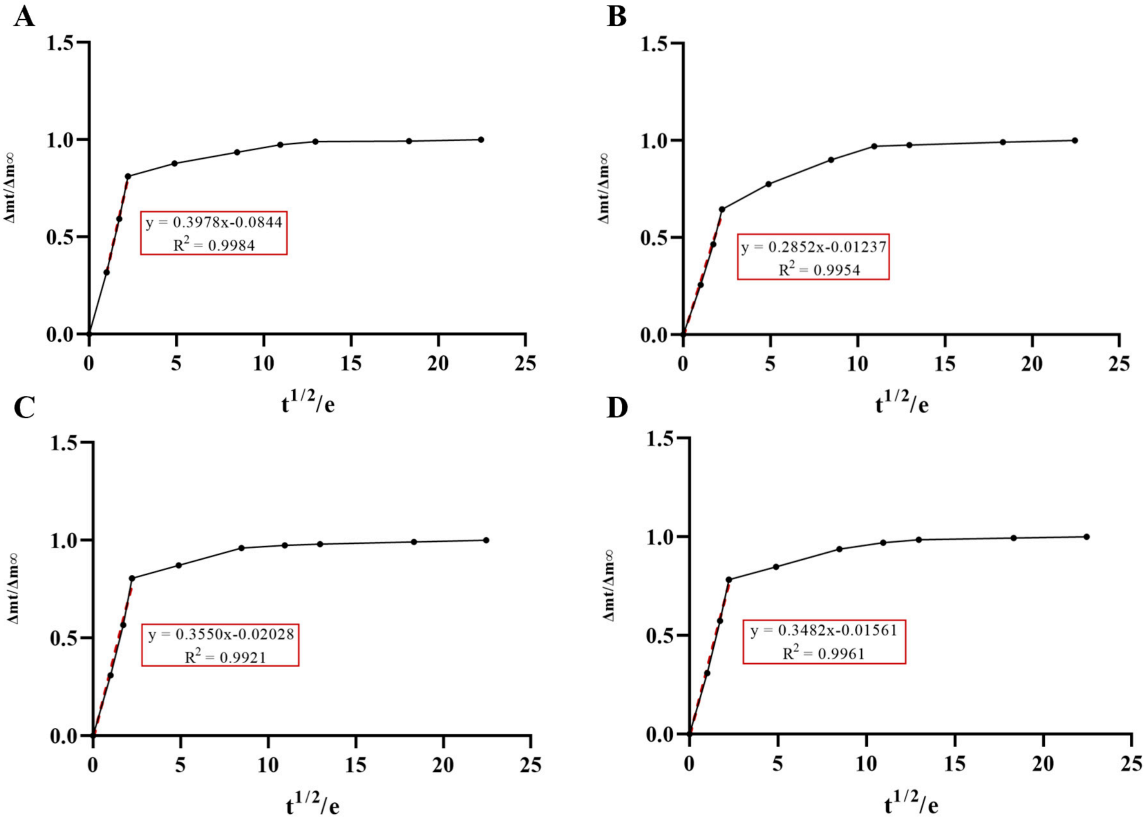

Figure S1. Time dependence of the diffusion coefficient calculated for HA films with different DVS and GO content with the kinetic model proposed by Fick's law: (a) HA-DVS1, (b) HA-0.1GO-DVS1, (c) HA-0.2GO-DVS1 and (d) HA-DVS2.

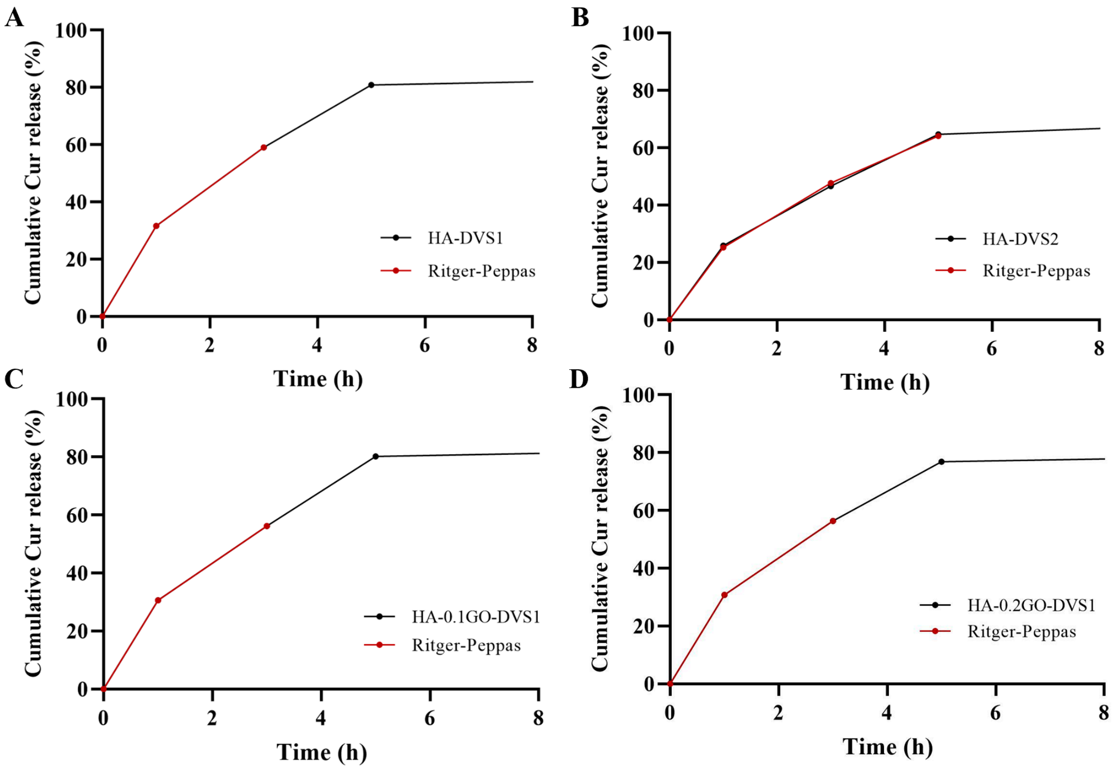

Figure S2. Curcumin release data fitted to Ritger-Peppas' model: (A) HA-DVS1, (B) HA-DVS2, (C) HA-0.1GO-DVS1 and (D) HA-0.2GO-DVS1.
